# Semantic Relations: Extending SNOMED CT and Solor

**DOI:** 10.1055/a-2606-9411

**Published:** 2025-10-03

**Authors:** Melissa P. Resnick, James Hitt, Wilmon McCray, Kendria Hall, Frank LeHouillier, Steven H. Brown, Keith E. Campbell, Diane Montella, Jonathan Nebeker, Peter L. Elkin

**Affiliations:** 1 Department of Biomedical Informatics, University at Buffalo, Buffalo, New York, United States; 2 U.S. Department of Veterans Affairs, Western New York Health Care, Buffalo, New York, United States; 3 U.S. Department of Veterans Affairs, Office of Clinical Informatics, Washington, District of Columbia, United States; 4 Faculty of Engineering, University of Southern Denmark, Odense, Denmark

**Keywords:** SNOMED CT, clinical decision support, natural language processing, semantics, patient care

## Abstract

**Background:**

Terminologies, such as Systematized Nomenclature of Medicine Clinical Terms (SNOMED CT) and Solor, assist with knowledge representation and management, data integration, and triggering clinical decision support (CDS) rules. Semantic relations in these terminologies provide explicit meaning in compositional expressions, which assist with many of the above-listed activities.

**Objective:**

The aims of this research are to: (1) identify semantic relations that are not fully present in SNOMED CT and Solor and (2) use these identified semantic relations with terms that are currently present in SNOMED CT and Solor to form triples.

**Methods:**

We identified relations that were not fully present in either SNOMED CT or Solor and were important for VA Knowledge Artifacts (KNARTS). These terms and the relations were formed into triples. The relations, terms, classifications, and sentences were used to implement the relations in the High Definition-Natural Language Processing (HD-NLP) program.

**Results:**

There are a total of 38 semantic relations. These had use cases built for each and were implemented in the Solor HD-NLP server for tagging of KNARTS.

**Conclusions:**

These new SNOMED CT and Solor semantic relations will give clinicians the ability to add more detail and meaning to their clinical notes. This can improve our ability to trigger CDS rules, leading to improved CDS provided to clinicians during patient care.

## Background and Significance

Throughout the decades, terminologies have played an important role in informatics. They serve many purposes: (1) knowledge management, (2) data integration, and (3) decision support, just to name a few. 1 Three of the most commonly used terminologies are Systematized Nomenclature of Medicine Clinical Terms (SNOMED CT), Logical Observations Identifiers Names and Codes (LOINC), and RxNorm. 2 There has been increasing interest in combining these terminologies, as they would provide enhanced expressivity and interoperability. 1 Solor is one such example.

### Solor

According to Resnick et al, Solor is an integrated terminology system created in collaboration with the U.S. Veterans Affairs (VA), which combines SNOMED CT (representing diseases, findings, and procedures), LOINC (representing laboratory test results), and RxNorm (representing medications). 1 Thus, Solor builds upon SNOMED CT, RxNorm, and LOINC by integrating their content and semantics. 1 3 4 In this paper, we discuss the semantic relations and the further extension of this portion of Solor.

### Purpose of Semantic Relations

Semantic relations serve at least three purposes: (1) making semantics, or meaning, explicit; (2) enabling automatic classification; and (3) allowing postcoordination. 5 6 First, semantic relations provide a formal way to express the meaning of concepts. 5 6 This is especially important in terminologies such as SNOMED CT, as meaning is typically not expressed through free-text definitions. 5 In fact, as of 2018, there were only 4,000 definitions for 341,000 active concepts. 2

Second, based on definitions of concepts, a reasoning or inference engine can perform automated classification. 5 6 For example, the reasoning engine can infer that a “fracture of femur” is also a type of “fracture of bone,” which is, in turn, a type of “disease.”

Third, postcoordination provides for the construction of detailed concepts without the need to have every concept represented in a terminology. 5 6 Postcoordination is valuable for clinical users who want to provide detailed information about a patient. 5 6 It is also useful for modelers who maintain or develop extensions for terminologies such as SNOMED CT. 5 6

### SNOMED CT Relations

SNOMED CT is built on a description logic model. 4 7 8 There are three components to this model: Concepts, Descriptions, and Relations. 7 Every concept is defined as a medically related idea, such as heart attack, which is referenced by a numeric identifier, “22298006.” 1 7 The description, or second component, is the textual component describing the concept. 7 In other words, it is the human-readable form of the concept. 7 Two descriptions are given to each identifier: the fully specified name (FSN), and the synonym. 7 The FSN represents a unique description of a concept's meaning. 7 For “22298006” the FSN is “myocardial infarction (disorder).” The synonym represents a term or terms that can be used to display or choose a concept. 7 “Heart attack” and “MI” are synonyms for “myocardial infarction (disorder).” The third component is the relations. 7 SNOMED CT has two main relations: “is_a,” and attributes. 3 9

The “is_a” relations organize the SNOMED CT concepts into 19 hierarchies, such as “body structure,” “procedure,” and “clinical finding.” 1 9 Almost all of the concepts have at least one “is_a” relationship, except for the top-level concept, which is the root concept and the most general. 9 Thus, due to the “is_a” relationships, one can traverse the hierarchies from the general concepts to more specific concepts at the bottom of the hierarchies.

There is a second type of relation, the attribute. 3 4 In SNOMED CT, the attribute (relation type) allows for the representation of a characteristic of a concept. 9 This provides more meaning to the concept. 10

SNOMED CT utilizes more than 50 attributes. 9 Some examples of attributes include: (1) associated morphology; (2) due to; and (3) causative agent. 11 The domain or source is the hierarchy to which a specific attribute can be applied, while the range or target is the set of concepts that are allowed as the value of the specific attribute. 9 For example, the domain for the “associated morphology” attribute is the “clinical finding” hierarchy. 3 9 The range for this same attribute is the concept “morphologically abnormal structure” and its subtypes or descendants. 9 Attributes are used with the following nine hierarchies: (1) clinical finding; (2) procedure; (3) evaluation procedure; (4) specimen; (5) body structure; (6) pharmaceutical/biologic product; (7) situation with explicit context; (8) event; and (9) physical objects. 9

### Solor Semantic Relations

Similar to SNOMED CT, Solor is built on a description logic model. 1 It utilizes both the “is_a” and the attribute relations. 3 4 Most of the concepts are shared by Solor and SNOMED CT and are arranged into hierarchies using the “is_a” relation. 1 3 The “is_a” relation also provides a mechanism to query and retrieve subtype concepts. 4

Currently, all of the SNOMED CT attributes have been incorporated into Solor. In 2019, one new attribute, “using device,” was added. 4 To improve the characterization and meaning of the concepts, approximately 40 additional attributes or semantic relations have been proposed and implemented. Before moving on to discuss new semantic relations, we will briefly discuss the increased flexibility that Solor can provide.

Solor can increase flexibility due to three of its characteristics. First, as mentioned above, it has a hierarchical structure. 1 This allows for detailed clinical data recording and retrieval. Second, through the use of its logic model, Solor provides different ways to convey the same or similar concepts. Finally, since Solor integrates SNOMED CT, LOINC, and RxNorm, it can improve the flow of clinical data among clinical documentation, decision support applications, and order entry at the point of care. 1

### New Solor Semantic Relations

The list of the new semantic relations came from the VA's work on semantic relations and the last author's work on the semantic relations from the structured product labels for drugs. One of the proposed semantic relations, “prevents,” is currently present as an unapproved attribute in SNOMED CT. 11 This means that it is not used in the SNOMED CT logic model. These semantic relations are being released as a Release Format 2 extension set to Solor.

Semantic relations (attributes) are normally implemented by adding them into the terminology. 3 4 This can be accomplished through addition of semantic relations to the logic model or by terminology extensions. 3 Instead of mapping an existing local code or term to a standard code, concepts would be directly represented using standard codes or logical expressions that conform to a description logic model. 3 On the other hand, terminology extensions can be employed. In this case, computable logical expressions based on SNOMED CT, LOINC, or RxNorm are created and added to extensions of these terminologies, which are then managed by an organization. 3 However, we chose to implement them via rules in the High Definition-Natural Language Processing (HD-NLP) program. 1 12 We employed the following steps for implementation.

The HD-NLP program uses a full semantic parse in memory followed by an encoder to link text to any set of ontologies preferred by the user for representing the knowledge in the free text. 1 Next, each entity is tagged as an affirmed, negative, or uncertain assertion. 1 We then automatically generate compositional expressions where applicable in the source text using the semantic relations available in the chosen ontologies. 1 We add the metadata from the record and link it to the information stored from computing over the input string. 1 HD-NLP uses several sources of synonymy, kept in separate synonym sets, which are available for interrogation to understand why certain results were obtained. 1 We made use of the HD-NLP to rapidly assign terminology concepts to text in patient records or Knowledge Artifact (KNART) text. 1

According to HL7, KNARTS are “structured, computable, and shareable representations of clinical knowledge.” 13 “Patients with diabetes should have regular comprehensive foot examination to identify risk factors predictive of ulcers and amputations” is an example of a clinical knowledge statement. 13 A KNART would then be used to represent this clinical knowledge statement and to integrate it into health care delivery systems, such as electronic health record systems, and clinical decision support (CDS). 13

The VA KNARTS are comprised of narrative clinical statements designed for CDS. 1 Each of these clinical statements were previously assigned Solor concepts by experienced human modelers. 1

## Objectives

Terminologies, such as SNOMED CT and Solor, assist with knowledge representation and management, data integration, and triggering CDS rules. Semantic relations provide explicit meaning in compositional expressions used for many of the above-mentioned activities. Thus, the aims of this research are to: (1) identify semantic relations that are not fully present in SNOMED CT and Solor and (2) use these identified semantic relations with terms that are currently present in SNOMED CT and Solor to form triples.

## Methods

Work on the KNARTS project at the VA was the impetus for the research on the new semantic relations. Thus, we identified relations that were not fully present in either terminology and were important for VA KNARTS. With the HD-NLP, we identified the semantic relations used in CDS and drug datasets. Once added to SNOMED CT and Solor, these semantic relations will be used to improve CDS rules.

First, each semantic relation was read one at a time to determine its meaning. Next, concepts were generated for the semantic relation. For example, the concepts “diabetes,” “heart disease,” and “obesity” were considered for the semantic relation “has_risk_factor.” These were formed into the triples (subject, verb, object): (1) heart disease “has_risk_factor” diabetes and (2) diabetes “has_risk_factor” obesity.

In the next step, the classifications for the subject and object of the semantic relation were determined, only some of which appear in the SNOMED CT hierarchies. This is because Solor incorporates three different terminologies.

For diabetes “has_risk_factor” obesity, the classification for diabetes is “disease;” the classification for obesity is “finding.” Care was taken to be sure to use each type of triple only once. In the case of “has_risk_factor,” disease “has_risk_factor” finding was employed only once.

The semantic relations with their concepts were given to physicians with instructions to write one sentence for each triple. They wrote sentences similar to those in their chart notes. For example: “Obesity is a risk factor for diabetes.” This represents the triple: diabetes “has_risk_factor” obesity.

In the final step, the semantic relations, concepts, classifications, and sentences were used to write rules for the HD-NLP program. These rules were written in the JSON programming language. The rules followed the general format, given a relation, the classifications of terms are the operand (come before the relation) and the terms become the specification (come after).

## Results

There is a total of 38 semantic relations with 68 discovered sets of terms and classifications. Of these: 23 relations have only 1 set; 6 relations have 2 sets; 5 relations have 3 sets; 2 relations have 4 sets; and 2 relations (“has_indication” and “prevents”) have 5 sets.

Of the 68 discovered subject/verb/object triples, there are 38 unique subject/object combinations ( Table 1 ). The top 3 are: medication/finding (10 out of 68), medication/disease (6 out of 68), and medical procedure/disease (4 out of 68). There are 13 unique subjects. The top 3 are: medication (33 out of 68), medical procedure (8 out of 68), and disease (6 out of 68). There are 16 unique objects. The top 3 are: finding (21 out of 68), disease (16 out of 68), and medication (7 out of 68).

**Table 1:** Preexisting semantic relations and discovered relational classifications and terms

Semantic relation	Relation with classifications	Example of terms with relation
Delays	Medication delays finding	Opioids delays gastric emptying
Exacerbates	Substance exacerbates finding	Pollen exacerbates allergic reaction
has_adverse_reaction	Medication has_adverse_reaction finding	Morphine has_adverse_reaction respiratory depression
has_adverse_reaction_action	Behavior has_adverse_reaction_action medication	Opioid overdose has_adverse_reaction_action Narcan
has_adverse_reaction_in_combination_with	Medication has_adverse_reaction_in_combination_with medication	Benzodiazepines has_adverse_reaction_in_combination_with opioids
has_adverse_reaction_in_population	Medication has_adverse_reaction_in_population age	First-generation antihistamines has_adverse_reaction_in_population elderly
has_adverse_reaction_in_population_by_age	Medication has_adverse_reaction_in_population_by_age elderly	Antipsychotics has_adverse_reaction_in_population_by_age older
has_adverse_reaction_in_population_by_gender	Medication has_adverse_reaction_in_population_by_gender female	Rosiglitazone has_adverse_reaction_in_population_by_gender female
has_adverse_reaction_in_population_by_preexisting_condition	Medication has_adverse_reaction_in_population_by_preexisting_condition disease	Nonselective beta blockers has_adverse_reaction_in_population_by_preexisting_condition reactive airway disease
has_adverse_reaction_in_population_by_race_ethnicity	Asian has_adverse_reaction_in_population_by_race_ethnicity finding	Asian race has_adverse_reaction_in_population_by_race_ethnicity anticoagulant-related ADEs
has_adverse_reaction_in_population_pregnancy	Behavior has_adverse_reaction_in_population_pregnancy disease	Drinking alcohol has_adverse_reaction_in_population_pregnancy fetal alcohol syndrome
has_adverse_reaction_severity	Medication has_adverse_reaction_severity behavior	Benzodiazepines has_adverse_reaction_severity falls
	Medication has_adverse_reaction_severity finding	Benzodiazepines has_adverse_reaction_severity cognitive impairment
has_associated_lab_test_result	Disease has_associated_lab_test_result lab result	Diabetes mellitus has_associated_lab_test_result hyperglycemia
	Finding has_associated_lab_test_result lab result	Anemia has_associated_lab_test_result low hemoglobin
	Infection has_associated_lab_test_result lab result	Bacterial infection has_associated_lab_test_result leukocytosis
has_black_box_warning	Medication has_black_box_warning lab result	Clozapine has_black_box_warning agranulocytosis
has_black_box_warning_severity	Medication has_black_box_warning_severity behavior	Opioids has_black_box_warning_severity abuse
	Medication has_black_box_warning_severity finding	Opioids has_black_box_warning_severity addiction
has_contraindication	Finding has_contraindication medication	G6PD deficiency has_contraindication sulfa drugs
has_contraindication_in_combination_with	Medication has_contraindication_in_combination_with medication	Demerol has_contraindication_in_combination_with MAO inhibitors
has_contraindication_in_population	Medical procedure has_contraindication_in_population coagulopathy	Neurosurgery has_contraindication_in_population patients with coagulopathy
	Medical procedure has_contraindication_in_population Rh-negative	Transfusion Rh-positive has_contraindication_in_population patients with Rh-negative blood
	Medication has_contraindication_in_population anticoagulation	NSAIDs has_contraindication_in_population patients on anticoagulation therapy
has_contraindication_in_population_by_age	Medication has_contraindication_in_population_by_age neonate	Chloramphenicol has_contraindication_in_population_by_age 0–28 d
has_contraindication_in_population_by_gender	Medication has_contraindication_in_population_by_gender male	Oxytocin has_contraindication_in_population_by_gender men
has_contraindication_in_population_by_preexisting_condition	Radiological test has_contraindication_in_population_by_preexisting_condition pacemakers	MRI has_contraindication_in_population_by_preexisting_condition patients with pacemakers
has_contraindication_in_population_pregnancy	Medication has_contraindication_in_population_pregnancy disease	Thalidomide has_contraindication_in_population_pregnancy birth defects
has_indication	Lab result has_indication disease	High HgA1C has_indication type 2 diabetes mellitus
	Medical procedure has_indication disease	Cholecystectomy has_indication cholecystitis
	Medical procedure has_indication finding	Thoracentesis has_indication pleural effusion
	Medication has_indication disease	Metformin has_indication type 2 diabetes mellitus
	Medication has_indication infection	Antibiotics has_indication bacterial infection
has_indication_for_prevention	Medical procedure has_indication_for_prevention disease	Prophylactic mastectomy has_indication_for_prevention breast cancer
	Medication has_indication_for_prevention finding	Imdur has_indication_for_prevention angina
has_indication_for_prevention_primary	Medical procedure has_indication_for_prevention_primary disease	Colonoscopy has_indication_for_prevention_primary colon cancer
	Nutrition has_indication_for_prevention_primary disease	Calcium and vitamin D has_indication_for_prevention_primary osteoporosis
has_indication_for_prevention_ secondary	Medical device has_indication_for_prevention_ secondary finding	AICD has_indication_for_prevention_ secondary cardiac death
	Medical procedure has_indication_for_prevention_ secondary finding	Cardiac stent placement has_indication_for_prevention_ secondary heart attack
	Medication has_indication_for_prevention_ secondary finding	Corticosteroids has_indication_for_prevention_ secondary asthma attack
has_indication_for_treatment	Medical device has_indication_for_treatment finding	CPAP has_indication_for_treatment sleep apnea
	Medical procedure has_indication_for_treatment disease	Surgery has_indication_for_treatment lumbar stenosis
	Medication has_indication_for_treatment disease	Insulin has_indication_for_treatment type 1 diabetes mellitus
has_indication_for_treatment_adjuvant	Medication has_indication_for_treatment_adjuvant disease	Chemotherapy has_indication_for_treatment_adjuvant cancer
	Medication has_indication_for_treatment_adjuvant finding	Antidepressants has_indication_for_treatment_adjuvant chronic pain
has_risk_factor	Disease has_risk_factor behavior	Lung cancer has_risk_factor smoking
	Disease has_risk_factor disease	Heart disease has_risk_factor diabetes
	Disease has_risk_factor finding	Diabetes has_risk_factor obesity
has_treatment	Disease has_treatment behavior	Depression has_treatment psychotherapy
	Disease has_treatment medication	Diabetes has_treatment metformin
	Finding has_treatment medication	Headache has_treatment acetaminophen
	Infection has_treatment medication	*Streptococcus pneumoniae* has_treatment antibiotic
Increases	Bacteria increases finding	*Streptococcus pneumoniae* increases body temperature
	Behavior increases finding	Alcohol use increases liver damage
	Finding increases lab result	Uncontrolled diabetes increases A1C
	Medication increases finding	Adrenaline increases heart rate
is_anesthetic_for	Medication is_anesthetic_for finding	Lidocaine is_anesthetic_for pain
Prevents	Behavior prevents disease	Exercise prevents osteoporosis
	Behavior prevents finding	Exercise prevents obesity
	Medication prevents disease	MMR vaccine prevents measles mumps and rubella
	Medication prevents finding	Motrin prevents dysmenorrhea
	Nutrition prevents disease	Dietary calcium prevents osteoporosis
Prolongs	Finding prolongs procedure	Vitamin K deficiency prolongs prothrombin time
	Medication prolongs procedure	Coumadin/warfarin prolongs prothrombin time
requires_regular_monitoring	Medication requires_regular_monitoring procedure	Coumadin requires_regular_monitoring PT/INR
requires_regular_monitoring_action	Medication requires_regular_monitoring_action procedure	Ketamine infusion requires_regular_monitoring_action LFTs
requires_regular_monitoring_threshold	Medication requires_regular_monitoring_threshold lab result	Vancomycin requires_regular_monitoring_threshold 10–15 µg/mL
Suppresses	Medication suppresses finding	Codeine suppresses cough

Abbreviations: ADE, adverse drug event; AICD, automatic implantable cardioverter–defibrillator; CPAP, continuous positive airway pressure; LFT, liver function test; MAO, monoamine oxidase; MMR, Measles, Mumps, and Rubella; MRI, magnetic resonance imaging; NSAIDs, nonsteroidal anti-inflammatory drugs; PT/INR, prothrombin time/International Normalized Ratio.

## Discussion

One of the contributions of this work are the triples. These triples can be leveraged with at least two informatics tools: (1) Natural Language Processing (NLP) tools and (2) CDS tools. 14 In the case of NLP, the triples provide increased accuracy in tagging the unstructured text, which can influence other activities. 14 Triples also allow for the triggering of CDS rules, which in turn, can assist clinicians while caring for their patients. 14 Semantics or attributes are one of the important components of these triples, which will be discussed below.

The discussion about the KNARTS led to these new semantic relations. For example, conversations with respect to risk of falls and prevention of falls led to the semantic relations “has_risk_factor” and “prevents,” respectively. Other discussions about glucose monitoring and glucagon injections led to the creation of “requires_regular_monitoring” and “has_treatment,” respectively. Through this process, the remaining semantic relations were developed.

Other semantic relations worth discussing involve “has_indication_for_prevention,” “has_indication_for_prevention_primary,” and “has_indication_for_prevention_secondary.” With these semantic relations, the organizing concept is “has_indication_for_prevention.” The concept “has_indication_for_prevention_primary” refers to prevention before the development of a disease. Finally, “has_indication_for_prevention_secondary” entails prevention after one has a disease. This group of semantic relations provides additional richness to SNOMED CT and Solor. This richness allows clinicians to be more expressive while writing their notes.

One of the goals of this research was to provide as many examples for each semantic relation as possible. This allows for the discovery of the most real-world applications. In this way, improvements can be made to SNOMED CT and Solor, leading to increased usability by clinicians. In addition, these real-world cases can then be used for CDS rules

Much like SNOMED CT, the Solor classifications for the new semantic relations act as a domain and a range. In some instances, the classifications can both be in SNOMED CT. In the case of diabetes “has_risk_factor” obesity, the domain, or classification, is disease and the range, or classification, is finding. However, in some cases, only one classification is present in SNOMED CT. In the case of first-generation antihistamines (classification medication) “has_adverse_reaction_in_population” elderly (classification age), the classification “medication” is in RxNorm, whereas the classification “age” is in SNOMED CT. Thus, the new Solor semantic relations and their classifications bring the three terminologies in Solor together.

The semantic relations also enhance the expressiveness of Solor. This is accomplished by adding terms for laboratory results and drugs, which are linked by the new semantic relations. Here, LOINC and RxNorm contribute new subjects and objects, which can be utilized with the semantic relations. This provides multiple ways to represent thoughts clinicians choose to convey in their chart notes.

Clinicians need ways to represent the meaning of their utterances. Semantic relations and SNOMED CT attributes allow them to better communicate the meaning of these utterances in their clinical notes. Likewise, these new semantic relations for SNOMED CT and Solor give clinicians additional methods for communicating this meaning.

The new SNOMED CT and Solor semantic relations can also play an important role in CDS. Here, the data from clinical notes and reports, with additional detail and meaning from the semantic relations, can then be used to trigger CDS rules. This, in turn, can improve the CDS provided to clinicians.

## Conclusion

In conclusion, it is believed that the new SNOMED CT and Solor semantic relations will allow for different ways to represent the detail and meaning in clinical notes. This will then improve CDS rules, and ultimately, the CDS provided to clinicians during patient care. As such, the semantic relations will enhance the utility of Solor.

In the future, we will test these new semantic relations with the HD-NLP system using clinical text. Finally, these semantic relations will be submitted to SNOMED CT for consideration for inclusion in the main standard.

## Clinical Relevance Statement

With the addition of the new semantic relations to the SNOMED CT and Solor terminologies, clinicians will be able to express their thoughts in their notes in more detailed and meaningful ways. The data from these notes can then be used to trigger CDS rules. Both of these uses of the new semantic relations can assist clinicians in providing improved care to their patients.

## Multiple-Choice Questions

1. What ability do semantic relations give clinicians?
  - Talk to their patients
  - Add more detail and meaning to their clinical notes
  - Read laboratory reports
  - Talk with other health care providers
  **Correct Answer** : The correct answer is option b. The semantic relations provide multiple ways to represent thoughts clinicians choose to convey in their chart notes. They allow clinicians to be more specific with these representations. Clinicians also need ways to represent the meaning of their thoughts. Semantic relations allow them to better communicate the meaning of these thoughts in their clinical notes. These more detailed and meaningful notes allow different members of the health care team to better understand the care provided to and needed by the patient.
2. What effect do the semantic relations have on patient care?
  - Decrease it
  - Impair it
  - Increase it
  - Improve it
  **Correct Answer** : The correct option is d. Semantic relations help to improve patient care. First, they allow clinicians to give more detail and meaning to their chart notes. Second, the data from these more detailed and meaningful notes can then be used to trigger clinical decision support rules. Finally, these two functions of semantic relations come together to improve patient care.
